# Dietary folic acid addition reduces abdominal fat deposition mediated by alterations in gut microbiota and SCFA production in broilers

**DOI:** 10.1016/j.aninu.2022.08.013

**Published:** 2022-09-25

**Authors:** Yanli Liu, Jiantao Yang, Xiaoying Liu, Rui Liu, Yibin Wang, Xinhuo Huang, Yingge Li, Ruifang Liu, Xiaojun Yang

**Affiliations:** a College of Animal Science and Technology, Northwest A&F University, Yangling 712100, China; b Nano Vitamin Engineering Research Center of Shaanxi Province, Xi'an 710000, China; c Shaanxi Province Animal Husbandry Technology Extension Station, Xi'an 710016, China

**Keywords:** Folic acid, Caecal microbiota, Abdominal adipocytes, Short chain fatty acid, Broilers

## Abstract

Intensive selective breeding for high growth rate and body weight cause excess abdominal fat in broilers. Gut microbiota and folic acid were reported to regulate lipid metabolism. A total of 210 one-day-old broilers were divided into the control (folic acid at 1.3 mg/kg) and folic acid groups (folic acid at 13 mg/kg) to illustrate the effects of folic acid on growth performance, abdominal fat deposition, and gut microbiota, and the experiment lasted 28 d. Results revealed that dietary folic acid addition decreased abdominal fat percentage (*P* < 0.05) and down-regulated genes expression related to cell proliferation and differentiation in abdominal fat including *IGF1, EGF, C/EBPα, PPARγ, PLIN1, FABP4* and *PCNA* (*P* < 0.05). Folic acid addition decreased caecal Firmicutes-to-Bacteroidetes ratio (*P* < 0.01) and increased the proportions of *Alistipes*, *Oscillospira*, *Ruminococcus*, *Clostridium*, *Dehalobacterium* and *Parabacteroides* (*P* < 0.05). Caecal acetic acid, and propionic acid contents were found to be higher under folic acid treatment (*P* < 0.05), which were negatively related to genes expression associated with adipocyte proliferation and differentiation (*P* < 0.05). *Ruminococcus* was positively correlated with caecal acetic acid content, and the same phenomenon was detected between propionic acid and *Oscillospira* and *Ruminococcus* (*P* < 0.05). Acetic acid and *Oscillospira* were identified to be negatively associated with abdominal fat percentage (*P* < 0.05). In conclusion, our data demonstrated that dietary supplementation of folic acid reduced fat deposition in broilers by inhibiting abdominal adipocyte proliferation and differentiation, which might be mediated by changes in gut microbiota and short chain fatty acid production.

## 1 Introduction

With the intensive breeding selection for growth rate and body weight in broiler chickens, the phenomenon of excess abdominal fat has followed which reduces feed conversion efficiency and entails huge economic loss. On the other hand, because of our propensity to overeat and lack of physical exercise, obesity has become widespread in humans in all stages of life. The broiler chicken has been considered an attractive biomedical model for eating disorders and obesity in humans based on its similarity in de novo lipid synthesis (Wang et al., 2017a). Therefore, how to reduce abdominal fat deposition is a topic of interest to many researchers.

Many works have emphasized the potential advantages of folic acid supplementation in lipid metabolism and obesity prevention. Li et al. (2018) implied that folic acid intervention ameliorated insulin resistance and reduced fat mass in obese mice by decreasing *ADCY3* and *RAPGEF4* methylation. Yu et al. (2014) also reported that folic acid caused a dose-dependent decrease in *PPARγ*, C/*EBPα* and *FAS* mRNA levels by changing DNA methylation of gene promoter regions during pre-adipocyte differentiation in chickens. Dietary folic acid addition could improve liver and spleen weight of weaned piglets (Wang et al., 2021) and attenuate high-fat diet-induced steatohepatitis in rats through ameliorating hepatic one-carbon metabolism, restoring the diversity of gut microbiota, and increasing deacetylase *SIRT1*-dependent *PPARα* levels (Xin et al., 2020). Mardinoglu et al. (2018) suggested that dietary intervention could promote rapid microbial shifts toward folate production, increase circulating folate concentration and finally, relieve non-alcoholic fatty liver disease. Gut microbiota have been found to modulate one-carbon metabolism and alter host folate and choline levels, impacting fat accumulation (Radziejewska et al., 2020). These findings support the beneficial role of folic acid in lipid metabolism. On the other hand, short chain fatty acids (SCFAs) were reported to mediate gut microbiota alterations in low calorie diets; thus SCFA-producing bacteria may hold therapeutic potential for obesity (Alsharairi, 2021). Therefore, dietary intervention causing intestinal microbiota alterations has the potential to influence gut homeostasis and therefore improve health via its SCFA metabolites, a viewpoint that is gaining increased attention.

Wang et al. (2017a) put forward an idea that abdominal fat deposition regulation in chickens was challenging to modify through dietary intervention during the first week due to animal maturity and nutritional transition from lipid-rich yolk to dietary carbohydrate and protein, but that changes were relatively easy and detectable from 12 to 49 d of age. Our previous study demonstrated that continuous folic acid perfusion from 3 to 10 d of age decreased abdominal fat percentage in broilers at d 11 (Liu et al., 2019b). This discrepancy may be related to the route of nutrient delivery. Indeed, the perfusion method can ensure precise nutrient provision, however it is not convenient for application in broiler feeding. In the previous study, we found that folic acid inhibited hepatic de novo lipid synthesis and promoted lipid exportation (Liu et al., 2018, 2019b). But whether folic acid could exert this function on lipid regulation via gut microbiota is still unknown in chickens.

All present reports have indicated that an innate relationship might exist between folic acid, gut microbiota and abdominal fat deposition. However, the effect of folic acid on gut microbiota in broilers is also unknown, and further molecular mechanisms of their specific relationship are still unclear. Development of the caecal microbiota has been found to be completed in broiler chickens around the age of 28 d (Huang et al., 2018; Richards et al., 2019). Taken together, based on our previous findings regarding abdominal fat reduction via folic acid perfusion (Liu et al., 2019b), to address the problems mentioned above, we carried out the current study to illustrate the effects of dietary folic acid addition on growth performance, abdominal fat deposition, gut microbiota and SCFAs in broilers from 1 to 28 d of age.

## 2 Materials and methods

### 2.1 Animal ethics statement

All animal protocols were approved by the Animal Care and Use Committee of the College of Animal Science and Technology of the Northwest A&F University (Shaanxi, China).

### 2.2 Experimental design

A total of 210 one-day-old Arbor Acres broilers were randomly allotted into 2 groups with 7 replicates and diets were arranged as follows: control group with basal diet (folic acid at 1.3 mg/kg); folic acid group with folic acid at 13 mg/kg. The ingredients and nutrient levels of the basal diets are shown in Table 1, which referred to our previous study (Liu et al., 2019b). All birds were fed in double-layer wired battery cages with free access to water and mash feed at the Experimental Teaching Center of Animal Science in the Norwest A&F University. For the first week, the temperature was controlled at 34 to 36 °C and then decreased by 2 °C per week. The relative humidity was maintained at 55% to 65% for the first 2 weeks, then 45% to 55% from 3 to 4 weeks. The lighting schedule was 23 h with 30 to 50 lx for the first week, and then reduced by 2 h per week with 25 lx. The study lasted 28 d.

**Table 1:** Formulation and proximate composition of the basal diets (as-fed basis, %).

Item	Content
Ingredients	
Corn	62.00
Soybean meal	24.50
Corn powder	4.45
Corn bran	4.00
Limestone	1.35
Dicalcium phosphate	1.23
Premix^1^	1.00
L-Lysine sulphate (70%)	0.58
Vegetable oil	0.30
Sodium chloride	0.30
Choline chloride (50%)	0.10
Preservatives	0.10
L-Threonine	0.06
DL-Methionine	0.03
Total	100.00
Nutrient levels	
Metabolism energy, kcal/kg	2,810
Crude protein	18.51
Ca	1.00
Total P	0.59
Digestible P	0.35
Lysine	1.20
Methionine	0.43
Methionine + cysteine	0.76

1 The premix provided the following per kilogram of diets: vitamin A, 8.4 kIU; vitamin D, 3.0 kIU; vitamin E, 54.90 mg; vitamin K, 2.70 mg; vitamin B_1_, 1.93 mg; vitamin B_2_, 7.92 mg; vitamin B_6_, 4.70 mg; vitamin B_12_, 0.04 mg; niacin, 50.30 mg; folic acid, 1.30 mg; pantothenic acid, 15.73; biotin, 0.20 mg; manganese, 83.20 mg; zinc, 93.60 mg; iron, 122.4 mg; iodine, 0.40 mg; copper, 10.00 mg; selenium, 0.39 mg; cobalt, 0.15 mg.

### 2.3 Growth performance and sampling

At 14 and 28 d, feed intake and body weight were recorded for each replicate, then the average daily feed intake (ADFI), average daily gain (ADG) and feed conversion ratio (FCR) were calculated. One bird was selected from each replicate and killed by cervical dislocation and dissected. Breast muscle, leg muscle and abdominal fat were then removed and weighed and expressed as a percentage relative to BW (gram of organ/gram of BW × 100%). At 28 d, the duodenum and jejunum were resected and washed using cold PBS to remove chymus, then the intestines were opened longitudinally and the mucosa was collected. In addition, fresh caecum chymus was collected for subsequent microbiome and SCFA analyses. After sampling, mucosa, caecum chymus and abdominal fat were immediately frozen in liquid nitrogen and stored at −80 °C.

### 2.4 Abdominal fat morphology

At 28 d, the abdominal fat tissue block was fixed in 4% formaldehyde over 48 h for histological analysis including hematoxylin-eosin (H&E) and Oil Red O staining which was operated by Wuhan Servicebio technology Co., Ltd (Wuhan, China).

### 2.5 Gene expression

Total RNA was extracted from abdominal fat and intestinal mucosa separately using a TRIZOL reagent kit (Accurate Biology, Xi'an, China). Then cDNA synthesis from 1,000 ng RNA was performed based on the protocol of UEIris RT mix with DNase (US Everbright Inc., Nanjing, China). Gene expression of folic acid transport carriers and adipocyte proliferation and differentiation was quantified by RT-PCR. The assay was carried out using 2 × SYBR Green qPCR Master Mix (US Everbright Inc., Nanjing, China) on a Roche-LightCycler 96 instrument (Switzerland, Basel). Primer sequences used in the current study are listed in Table 2. Detail PCR reaction and calculation methods are detailed in our previous study (Liu et al., 2018).

**Table 2:** Forward and reverse primer sequences for PCR analysis.

Gene	Accession number	Primer sequences, 5′ to 3′	Product size, bp
*β-actin*	L08165	F: ATTGTCCACCGCAAATGCTTC	113
		R: AAATAAAGCCATGCCAATCTCGTC	
*PCFT*	NM001205066	F: CAGTACCTATGGGATCGGCTG	179
		R: GAGAAGAGCCCCACGAAGAA	
*RFC*	NM001006513	F: TTTCTGGTTCCCATCGCTATTTTC	205
		R: AGACCAGTGCCAGCAGTGAAAAGT	
*ELOVL6*	NM001031539	F: GGTGGTCGGCACCTAATGAA	169
		R: TCTGGTCACACACTGACTGC	
*FR*	XM015280910	F: CATCCAGGATATGTGCTTGTATGA	180
		R: CAGCCCTTGTGCCAGTTCTC	
*IGF1*	NW003763484	F: CTGGTTGATGCTCTTCAGTTCG	142
		R: AGCCTCCTCAGGTCACAACTCT	
*LPL*	NM205282	F: CCGATCCCGAAGCTGAGATG	186
		R: ACATTCCTGTCACCGTCCAC	
*PPARγ*	NM001001460	F: CCAAGGCAGCGGCAAAATAA	188
		R: GTGCCCATAAATGATGGCCTAA	
*C/EBPα*	NM001031459	F: GACATCTGCGAGAACGAGCA	154
		R: GCATGCCGTGGAAATCGAAA	
*TGFβ1*	NM001318456	F: TCCAATATGGTGGTCCGTGC	158
		R: AACCCCCAAAAAGGGAACCAT	
*TGFβ2*	NM001031045	F: TCATGCGCAAGAGGATCGAG	247
		R: TCGGGGTAAAAAGGCTGCAT	
*FABP4*	NM204290	F: GCCTGACAAAATGTGCGACC	130
		R: ATTAGGCTTGGCCACACCAG	
*KLF5*	XM040657735	F: AAAAGACGCATCCACTAC	296
		R: AACAGCCTCGGCAACAA	
*PCNA*	NM204170	F: CTGAGGGCTTCGACACCTAC	142
		R: AGAGCCAACGTATCCGCATT	
*PLIN1*	NM001127439	F: AAAGCCCATCCAGTCCCAAC	99
		R: CCCTGGAGGTTCTCATGTCC	
*EGF*	NM001001292	F: TACTGTTGACTTTGCCAGCCC	240
		R: AGTAGGAATGGTGCAGGGTC	

*PCTF* = proton coupled folate transporter; *RFC* = reduced folate carrier; *ELOVL6* = elongase of very long chain fatty acids family member 6; *FR* = folate receptor; *IGF* = insulin growth factor; *LPL* = lipoprotein lipase; *PPAR* = peroxisome proliferators-activated receptor; *C/EBP* = CCAAT-enhancer-binding proteins; *TGF* = transforming growth factor; *FABP* = fatty acid binding protein; *KLF* = Krüppel-like factor; *PCNA* = proliferating cell nuclear antigen; *PLINI* = perilipin; *EGF* = epidermal growth factor.

### 2.6 Caecal microbiome

Caecal microbiome DNA was extracted for quality detection by agarose gel electrophoresis, and bacterial V3 to V4 regions were amplified using the primer set (F: ACTCCTACGGGAGGCAGCA, R: GGACTACHVGGGTWTCTAAT). After PCR product purification, library construction was performed for sequencing analysis. Detailed methods about taxonomy classification, alpha and beta diversity and differential analysis were based on our published report (Li et al., 2021) and the standard protocols from Personalbio Technology Co. Ltd. (Shanghai, China).

### 2.7 Caecal short chain fatty acids

GC–MS spectrometry was used to detect caecal SCFA concentrations. Firstly, 0.3 g caecal contents were homogenized in cold normal saline and centrifuged at 10,000 × *g* for 10 min at 4 °C. The supernatant was obtained and mixed with metaphosphoric acid. After 4 h quiescence at 4 °C, the mixture was centrifuged at 10,000 × *g* at 4 °C for 15 min and crotonic acid was added to the supernatant. Finally, pre-treatment samples were placed in a meteorological bottle for GC–MS analysis. Parameters were set according to reported methods (Song et al., 2020). The acetic acid, propionic acid, isobutyric acid, butyric acid and valeric acid peaks were measured and their concentrations were calculated using the ratio of the peak area with the standard solution.

### 2.8 Statistical analysis

All data were expressed as the mean with standard error and statistical analysis was carried out by Student's t-test using SPSS 21.0 statistical software. Correlation analysis among SCFAs, abdominal fat percentage and bacteria was obtained by Pearson's correlation procedure. A probability value of *P* < 0.05 or *P* < 0.01 was considered to be statistically significant, which is indicated as follows: ∗*P* < 0.05, ∗∗*P* < 0.01.

## 3 Results

### 3.1 Growth performance

Effects of dietary folic acid addition on growth performance in broilers are shown in Table 3. No significant difference was found in BW, ADG, ADFI or FCR during 1 to 14 d, 14 to 28 d and 1 to 28 d (*P* > 0.05). At 14 and 28 d of age, dietary folic acid addition had no effect on leg and breast muscle percentage (*P* > 0.05), but abdominal fat percentage was remarkably lower in the folic acid group when compared with the control group (*P* < 0.01).

**Table 3:** Effects of dietary folic acid addition on growth performance in broilers.

Days	Item	Control	Folic acid	SEM	*P*-value
1 to 14 d	BW _d 14_, g	440.1	446.6	7.55	0.521
	ADFI, g	38.5	38.1	0.81	0.695
	ADG, g	31.4	31.9	0.57	0.586
	FCR	1.23	1.20	0.04	0.547
	Leg, %	10.7	11.3	0.31	0.126
	Breast, %	12.8	13.2	0.42	0.514
	Abdominal Fat, %	0.74∗∗	0.55	0.05	0.007
14 to 28 d	BW _d__28_, g	1,246.2	1,224.3	22.7	0.413
	ADFI, g	87.3	81.3	2.55	0.065
	ADG, g	57.5	55.2	1.64	0.231
	FCR	1.52	1.48	0.05	0.469
	Leg, %	13.3	13.3	0.21	0.871
	Breast, %	21.4	21.6	0.54	0.829
	Abdominal Fat, %	0.89∗∗	0.63	0.04	0.003
1 to 28 d	ADF, g	63.2	59.7	2.20	0.423
	ADG, g	42.8	41.8	0.99	0.338
	FCR	1.48	1.42	0.03	0.140

BW = body weight; ADFI = average daily feed intake; ADG = average daily gain; FCR = feed conversion ratio.

The double asterisks (∗∗) denote statistically significant within a row (*P* < 0.01).

### 3.2 Gene expression of folic acid transport carriers in the intestine

**Fig. 1:**
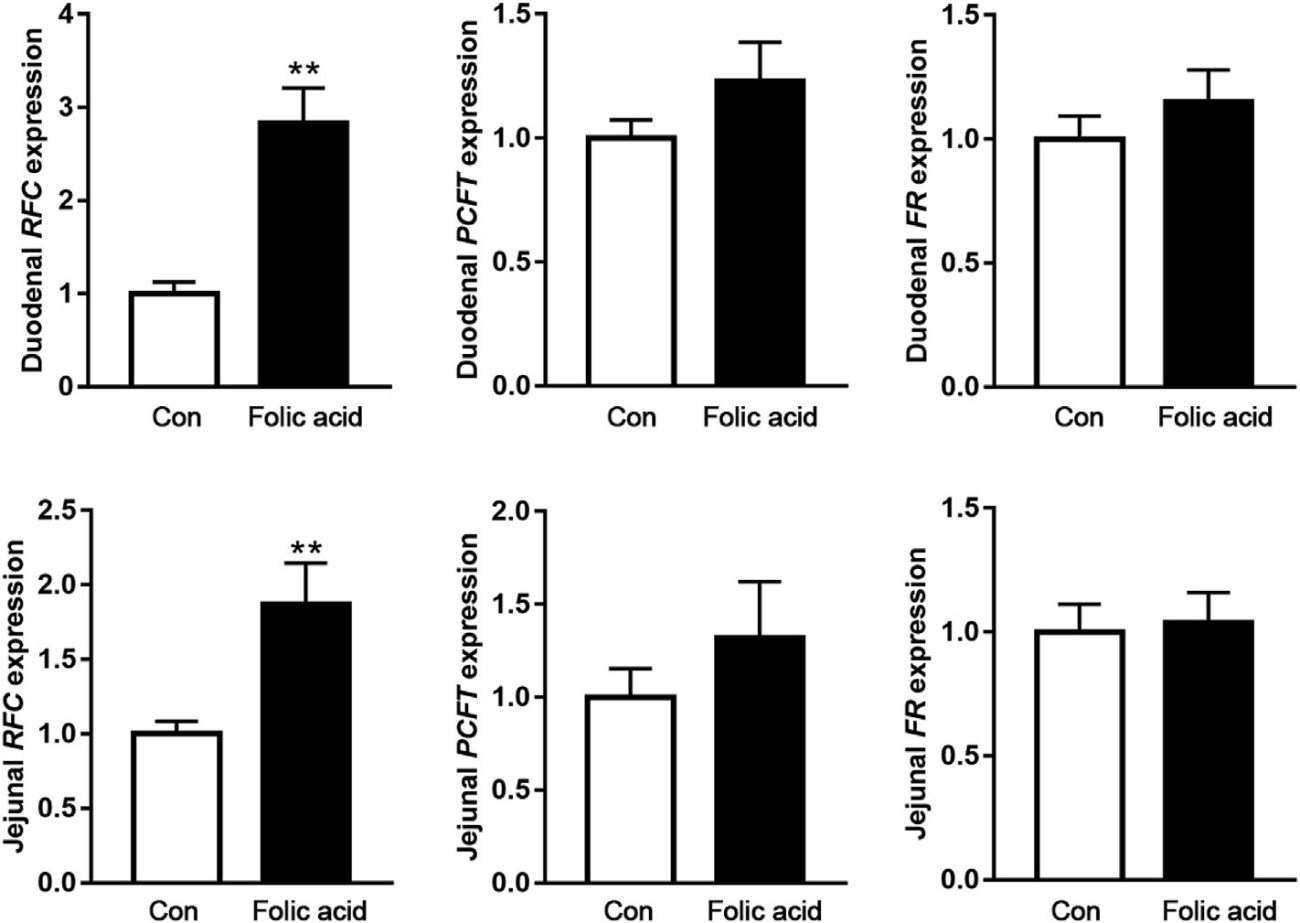
The effects of dietary folic acid addition on gene expression related to folic acid transport and absorption in duodenum and jejunum. Data are expressed as mean ± SEM (*n =* 7). The asterisk indicates statistically significant differences (two-tailed unpaired *t*-test, ∗*P* < 0.05, ∗∗*P* < 0.01). *PCFT =* proton coupled folate transporter; *RFC =* reduced folate carrier; *FR =* folate receptor.

Folic acid is absorbed mainly in the duodenum and proximal jejunum. Gene expression related to folic acid transport and absorption was measured and exhibited in Fig. 1. Dietary folic acid addition significantly increased *RFC* expression in the duodenum and jejunum (*P* < 0.05) but had no effect on *PCFT* and *FR* mRNA abundance (*P* > 0.05).

### 3.3 Abdominal fat morphology

**Fig. 2:**
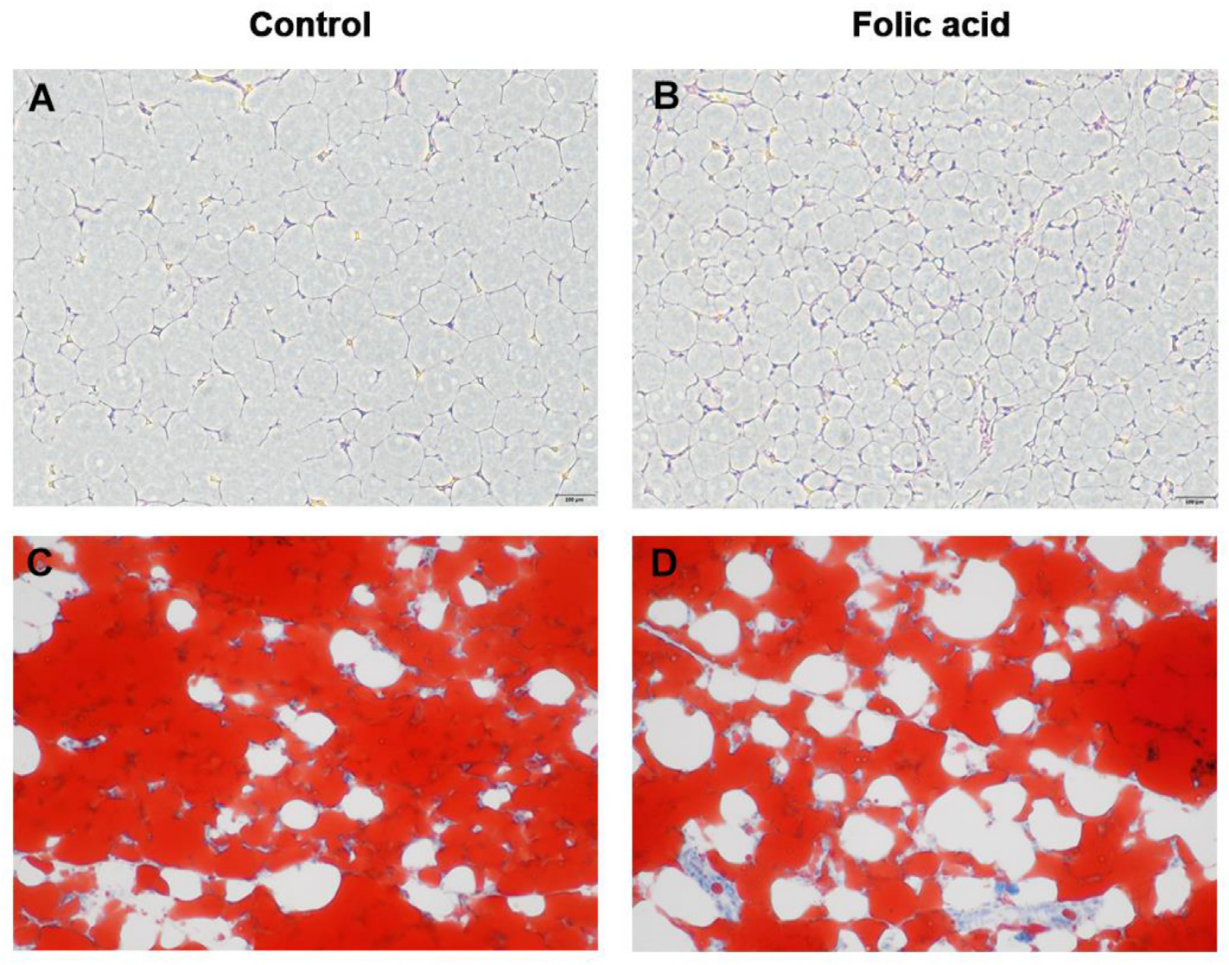
Morphological analysis of abdominal fat tissue (A and B) H&E staining of the control and folic acid groups, respectively (magnification 200× ; the scale bar is 100 μm), and white cavities are adipocytes (C and D) represent Oil Red O of the control and folic acid groups, respectively (magnification 200× ), and red circle drops indicate lipid droplets.

To understand how folic acid contributes to abdominal fat reduction, we performed an abdominal fat morphology analysis. As displayed in Fig. 2, H&E staining showed that adipocyte area and diameter were greater in the control group (Fig. 2A) than in the folic acid group (Fig. 2B). Similarly, Oil Red O staining also revealed that folic acid addition reduced lipid content of abdominal fat when compared with the control group (Fig. 2C).

### 3.4 Gene expression related to cell proliferation and differentiation in abdominal fat

**Fig. 3:**
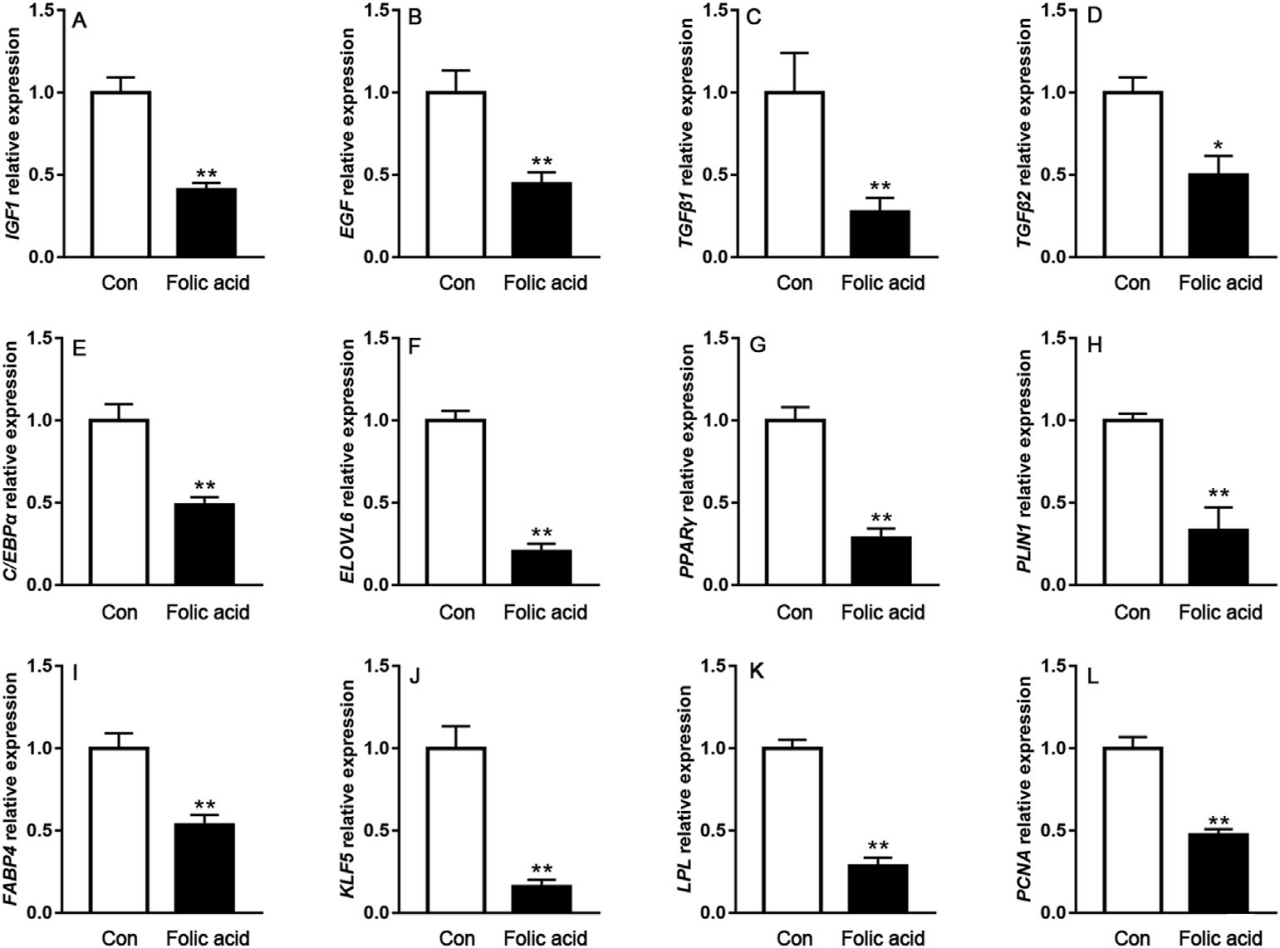
The effects of dietary folic acid addition on gene expression associated with adipocyte proliferation and differentiation. Data are expressed as mean ± SEM (*n =* 7). The asterisk indicates statistically significant differences (two-tailed unpaired *t*-test, ∗*P* < 0.05, ∗∗*P* < 0.01).

Adipose tissue develops through both adipocyte hyperplasia and hypertrophy. We next sought to quantify the effect of folic acid supplementation on gene expression associated with adipocyte proliferation and differentiation. As shown in Fig. 3, dietary folic acid addition significantly down-regulated *IGF1*, *EGF*, *TGFβ1*, *TGFβ2*, *C/EBPα*, *ELOVL6*, *PPARγ*, *PLIN1*, *FABP4*, *KLF5*, *LPL* and *PCNA* mRNA abundance in abdominal fat tissue (*P* < 0.05), all of which were associated with adipocyte proliferation and differentiation, or lipid deposition.

### 3.5 Caecal microbiome

**Fig. 4:**
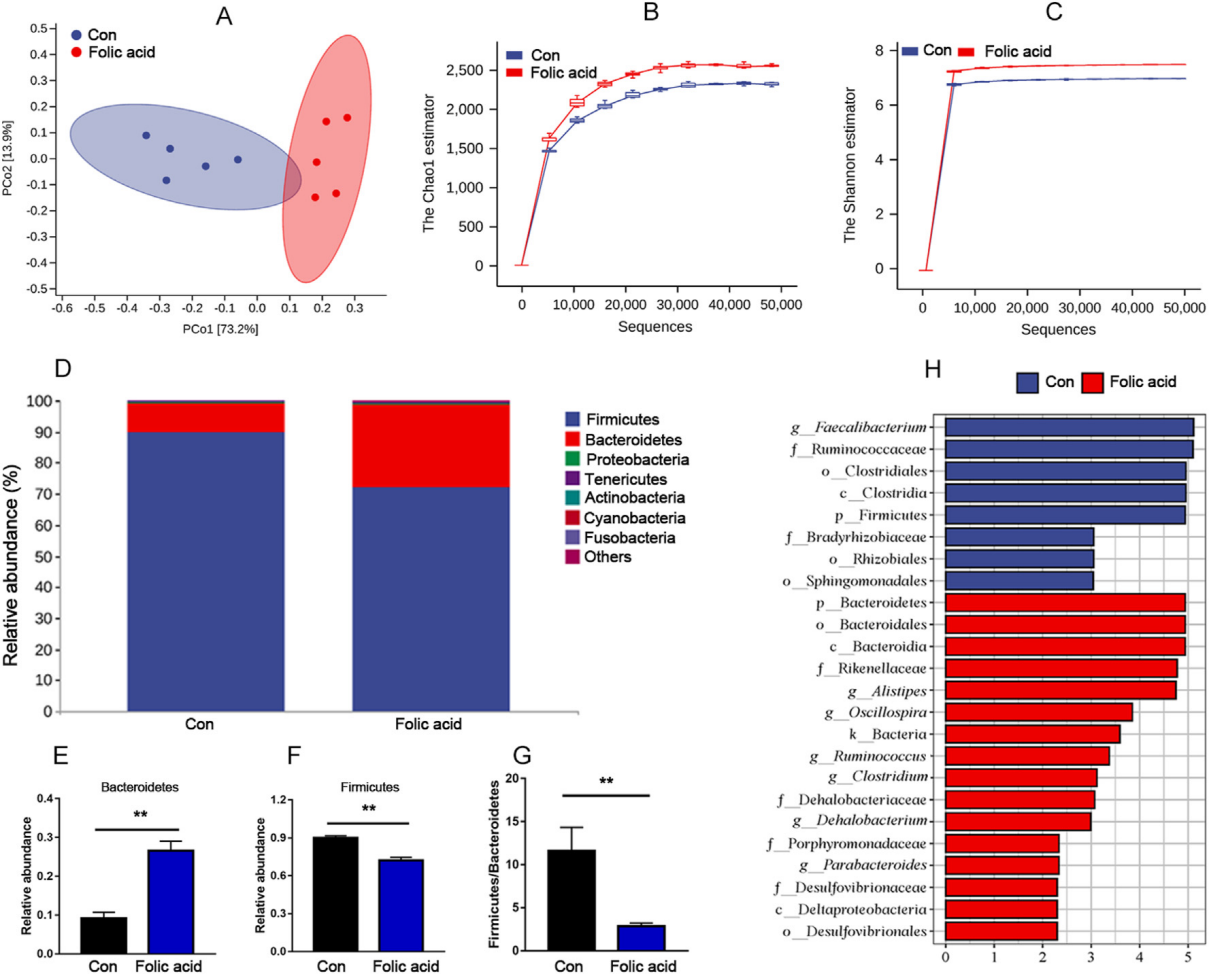
Caecal microbiome analysis (A) Principal coordinates analysis (PCoA) of caecal microbiome in broilers. Blue and red circles or points represent the control and folic acid group, respectively (B to C) Rarefaction curve of Chao 1 and Shannon index. Blue and red lines represent the control and folic acid group, respectively. (D) Relative abundance of bacterial composition in caecal contents at phylum level. Each color represents a different bacterium. The Y-axis shows the percentage of the bacteria (E to G) The effects of dietary folic acid addition on Firmicutes and Bacteroidetes relative content. Data are expressed as mean ± SEM (*n =* 5). The asterisk indicates statistically significant differences (two-tailed unpaired *t*-test, ∗*P* < 0.05, ∗∗*P* < 0.01). (H) Differential microbiota analysis based on LEfSe method. The default parameters were LDA score > 2 and *P* < 0.05). Bacteria with blue and red colors indicate higher levels in the control and folic acid groups, respectively. LDA = linear discriminant analysis.

As shown in Fig. 4A, principal coordinates analysis (PCoA) revealed significant differences in microbial communities between the control and folic acid groups. Rarefaction curve results also suggested that alpha diversity indices including Chao 1 and Shannon were higher in the folic acid group than the control group under the same sequencing depth (Fig. 4B to C). In order to compare the caecal microbiota composition and relative abundance, we firstly analyzed the species composition at the phylum level (Fig. 4D–G). Firmicutes and Bacteroidetes are the most abundant phyla; folic acid supplementation increased the percentage of Bacteroidetes while decreasing the relative abundance of Firmicutes (*P* < 0.01). Likewise, the ratio of Firmicutes to Bacteroidetes in the caecum was lower in the folic acid group (*P* < 0.01). Further, linear discriminant analysis Effect Size (LEfSe) analysis was used to identify differential microbiota between the control and folic acid groups. As exhibited in Fig. 4H, at the genus level, a higher abundance of *Alistipes*, *Oscillospira*, *Ruminococcus*, *Clostridium*, *Dehalobacterium* and *Parabacteroides* was found in the folic acid group (*P* < 0.05), while only *Faecalibacterium* was found to be higher in the control group (*P* < 0.05).

### 3.6 Caecal SCFA and correlation analysis

**Fig. 5:**
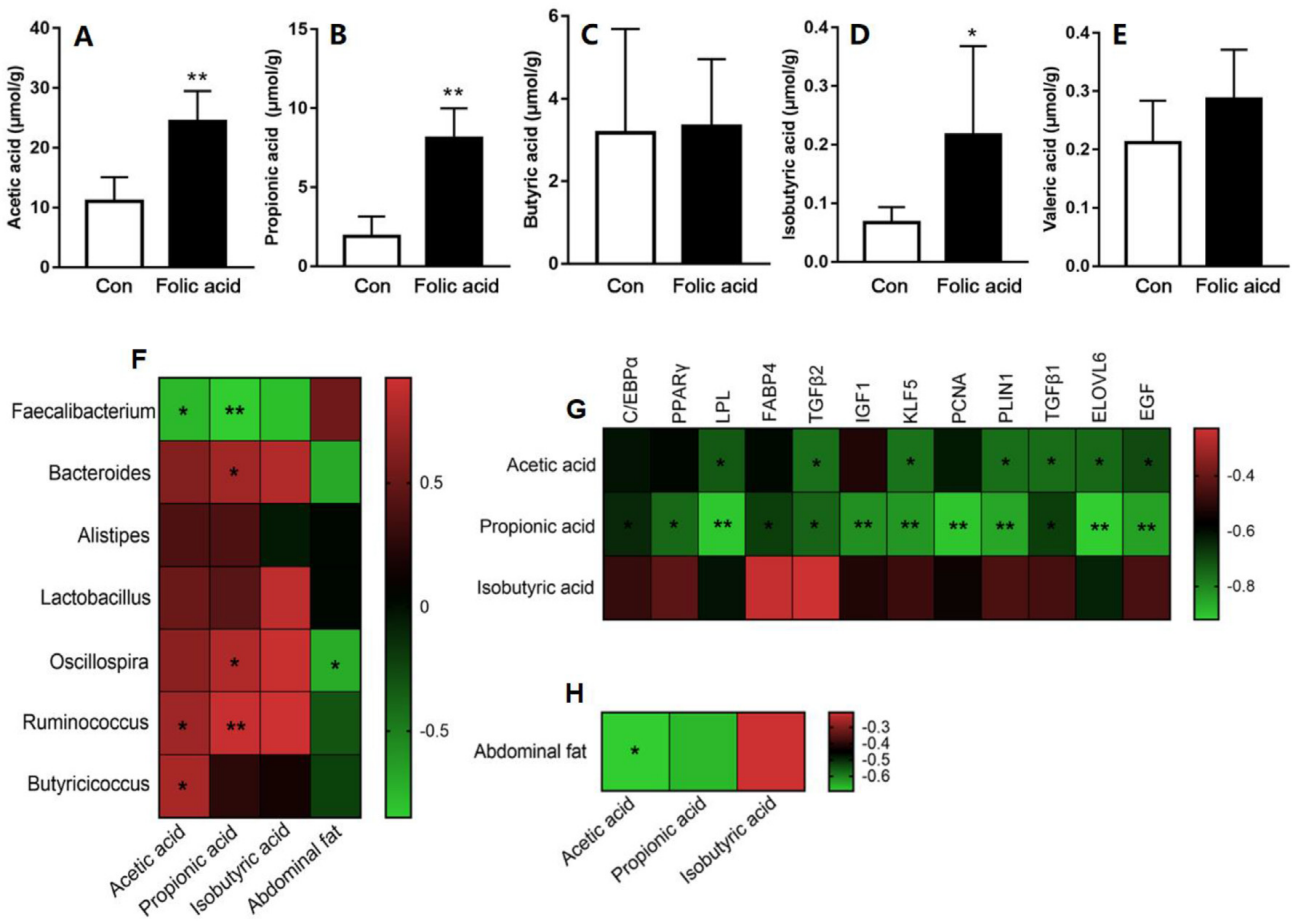
Caecal SCFA analysis. (A to E) The effects of dietary folic acid addition on caecal SCFA concentrations. Data are expressed as mean ± SEM (*n =* 7). (F to H) Heat map of correlation analysis among caecal SCFAs, caecal microbiota, gene expression, and abdominal fat percentage. The asterisk indicates statistically significant differences (two-tailed correlation analysis, ∗*P* < 0.05, ∗∗*P* < 0.01). Red and green colors represent positive and negative correlations, respectively, and color gradation indicates the size of the correlation coefficient.

To clarify the potential relationship between the caecal microbiome and abdominal fat phenotype, we also measured caecal SCFA concentration and carried out Pearson correlation analysis among caecal SCFA, caecal microbiota, abdominal fat percentage and gene expression in abdominal fat. As displayed in Fig. 5A–E, dietary folic acid addition increased acetic acid, propionic acid and isobutyric acid content (*P* < 0.05), but had no effect on butyric acid and valeric acid (*P* > 0.05). Meanwhile, as shown in Fig. 5F, *Faecalibacterium* was found to have significantly negative correlations with acetic acid and propionic acid (*P* < 0.05) whereas *Ruminococcus* and *Butyricicoccus* were positively correlated with caecal acetic acid content (*P* < 0.05). Positive relationships between propionic acid and *Oscillospira*, *Bacteroides* and *Ruminococcus* were also found (*P* < 0.05). Only *Oscillospira* was identified to be negatively associated with abdominal fat percentage among the 7 analyzed genera (*P* < 0.05). Furthermore, as exhibited in Fig. 5G, a significant negative correlation was found between acetic acid and *LPL*, *TGFβ1*, *TGFβ2*, *KLF5*, *PLIN1*, *ELOVL6* and *EGF* gene expression in abdominal fat (*P* < 0.05). Similarly, propionic acid was inversely related to the gene expression of all 12 genes associated with adipocyte proliferation and differentiation detected in the study. But as shown in Fig. 5H, amongst the 3 different SCFAs, only acetic acid was negatively correlated with abdominal fat percentage (*P* < 0.05).

## 4 Discussion

Abdominal fat is generally regarded as a waste product in meat chicken processing, which increases processing cost and decreases carcass yield. Wang et al. (2017a) summarized dietary factors that affect adipogenesis and adipose tissue expansion in broilers, such as fatty acids, carbohydrates, amino acids and probiotics. Vitamins, especially folic acid, have been linked to obesity or fatty liver disease (Miaaseth et al., 2021; Radziejewska et al., 2020). Our previous study found that folic acid perfusion reduced abdominal fat mass (Liu et al., 2019b), however the trial period was relatively short (from d 3 to 10) and the perfusion method is not suitable for intensive broiler feeding. Thus, the current study was carried out to examine the fat deposition-lowering function of folic acid via dietary intervention. On the other hand, most evidence supports that there exists a relationship among folic acid, gut microbiota and lipid metabolism (Radziejewska et al., 2020; Xin et al., 2020; Mardinoglu et al., 2018), which compels us to explore whether folic acid might derive its role in abdominal fat reduction through its interaction with gut microbiota.

Consistent with our previous study, the data in the current study indicated that intestinal folic acid absorption was enhanced and abdominal fat percentage was decreased in the folic acid group, suggesting that folic acid exerts a fat-lowing effect regardless of route of administration. There are 3 specific folic acid transporters in intestinal tract including *FR*, *RFC* and *PCFT*. Folic acid perfusion improved *FR* expression in the previous study (Liu et al., 2019b), while intestinal RFC expression was up-regulated in the folic acid group in the study. It seems that *FR* could ensure folic acid uptake efficiency under the condition of short-term high dose perfusion. Bai et al. (2021) also reported that dietary folic acid addition had no effect on intestinal *PCFT* expression but increased duodenal *RFC* mRNA abundance, which is consistent with our current findings. Thus, we speculate that the route of folic acid administration influences which transporter functions during the process of intestinal folic acid absorption.

Adipose tissue expansion originates from adipocyte formation and triacylglycerol accumulation inside lipid droplets (Wang et al., 2017a), which is positively correlated with abdominal fat mass. Abdominal fat morphology results showed that adipocyte area and diameter were smaller in the folic acid group when compared with the control group. Similarly, Oil Red O staining also revealed that folic acid addition reduced lipid content of abdominal fat, corresponding to the reduction of abdominal fat percentage. Guo et al. (2011) analyzed adipose tissue cellularity in chickens during the first 7 weeks and pointed out that adipocyte enlargement and cell number formation always occurred along with the age. *IGF1*, *EGF* and *TGFβ* promote adipocyte proliferation and differentiation. *PCNA* was considered as a proliferation marker. A previous study indicated that *KLF5* knockdown could inhibit primary pre-adipocyte proliferation (Wang et al., 2017b). *C/EBPα* and *PPARγ* are involved in terminal differentiation and are markers for lipid droplet appearance (Feve, 2005). In this study we found that folic acid significantly down-regulated the expression of these markers in abdominal fat. In avian species, *LPL* facilitated hydrolyzing lipoproteins from the liver and promoted fatty acid uptake in adipose tissue; then *FABP4* and *ELOVL6* contributed to adipocyte hypertrophy by mediating fatty acids for triglyceride synthesis (Wang et al., 2017). Correspondingly, lower mRNA levels of *LPL*, *FABP4* and *ELOVL6* were observed in abdominal fat from the folic acid group. Perilipin 1 (*PLIN1*), a lipid droplet associated protein in adipocytes, promotes chicken pre-adipocyte lipid accumulation (Sun et al., 2020). We found that *PLIN1* expression was down-regulated in abdominal fat by dietary folic acid addition. These results demonstrated that folic acid does indeed suppress adipocyte proliferation, differentiation and adipogenesis, which was in accordance with the findings from our previous folic acid perfusion study (Liu et al., 2019). All these observations support the results of abdominal fat morphology and its percentage reduction in this study.

An important finding in our present study was that dietary folic acid drives caecal microbiota dynamics. Folic acid supplementation increased the percentage of Bacteroidetes while decreased the relative abundance of Firmicutes, and affected the relative abundance of some microbiota at the genus level. It has been reported that unabsorbed folic acid enters the caecum and changes the caecal microbiota of laying hens (Bai et al., 2021). In fact, many works have reported the relationship between folic acid and the gut microbiome, for example, Liu et al. (2020) observed positive impacts of dietary folic acid and branched-chain volatile fatty acid addition on nutrient digestibility and microbiota composition responsible for fibre degradation in weaned calves. Wang et al. (2019) also reported that folic acid supplementation increased ADG, ruminal total VFA concentration and microbial growth in post-weaned dairy calves. Others have pointed out that rumen-protected folic acid improved nutrient digestibility and ruminal enzyme activity in dairy cows and bulls (Du et al., 2019; Wang et al., 2020), and also altered ruminal fermentation pattern to favour acetate and propionate production in dairy cows (Du et al., 2019). In the current study, dietary folic acid had no effect on BW, ADG and FCR in broilers but reduced abdominal fat percentage. Cornejo-pareja et al. (2019) reviewed the link between microbiota and fat mass. In the current study, folic acid addition increased the relative abundance of *Alistipes*, *Oscillospira*, *Ruminococcus*, *Clostridium*, *Dehalobacterium* and *Parabacteroides*, suggesting that gut microbiota modulations might contribute to abdominal fat reduction. Ley et al. (2006) investigated the relationship between gut microbial ecology and body fat in humans and found that obese people had fewer Bacteroidetes and more Firmicutes when compared with lean people, which was consistent with our results. Lower Firmicutes-to-Bacteroidetes ratio was found in the abdominal fat-lowering group in this study, indicating that gut microbiota changes might be one of mechanisms through which folic acid affects abdominal fat reduction.

Considering that gut microbiota elicits the regulatory function on host homeostasis via its metabolites, SCFAs, we also measured caecal SCFA contents. Results indicated that folic acid addition increased acetic acid, propionic acid and isobutyric acid content, and had no effect on butyric acid and valeric acid. Maternal methyl-donor addition containing folic acid increased the concentration of individual and total SCFAs in offspring piglets (He et al., 2021). Increased SCFAs may serve as an energy source for microbes and decrease energy utilization of amino acids or protein fermentation (Alsharairi, 2021; Du et al., 2019). Feeding imbalanced folic acid and choline diets during pregnancy could alter gut microbiota and SCFA concentration, thus causing the obesogenic phenotypes in rat offspring (Mjaaseth et al., 2021). Hernández et al. (2019) reported that acetate may act as a direct mediator and have important regulatory roles in weight control and insulin sensitivity. Acetic acid and propionic acid have been shown to inhibit lipid content in human adipocyte-types in diabetes, but had no effect on lipid accumulation in normal human adipocytes (Naraoka et al., 2018). Liu et al. (2019a) found that subcutaneous injection of acetate decreased triglyceride concentrations in plasma and adipocytes as well as absolute mass of adipose tissue, which was associated with *FAS* downregulation and *CPT1* upregulation in adipose tissue. Wang et al. (2021) found that folic acid addition could improve acetic acid and valeric acid content in the caecum and that *Lactobacillus* was positively correlated with acetic acid production.

In order to primarily examine the relationship among gut microbiota, SCFAs and abdominal fat deposition, Pearson correlation analysis was performed. On the one hand, the correlation analysis revealed that *Oscillospira* was negatively associated with abdominal fat percentage in the study. Although no significant relationship was found between the other bacteria and abdominal fat percentage, their SCFA metabolite might elicit effects on abdominal fat reduction. Mishra et al. (2020) reported that the function of *Enterococcus faecalis* AG5 in preventing obesity was attributed to its product, propionic acid, which played a role in adipocyte apoptosis. Among the different genera, *Faecalibacterium* was found to have significantly negative correlations with acetic acid and propionic acid. *Ruminococcus* and *Butyricicoccus* were positively correlated with caecal acetic acid content. Lower *Faecalibacterium* and higher *Ruminococcus* were found in the folic acid group, which conformed to caecal acetic acid improvement. On the other hand, significantly positive relationships between propionic acid and *Oscillospira*, *Bacteroides* and *Ruminococcus* were also determined, which supported the observed higher propionic acid content in the folic acid group. Song et al. (2019) had proved that oral sodium propionate reduced the mass of white adipose tissue and the mean area of adipocytes and restored gut microbiota dysbiosis induced by high-fat diets in mice. Similarly, oral sodium propionate reduced fat deposition in broiler chickens by decreasing adipocyte mean area and triglyceride content in abdominal fat (Li et al., 2021), which agrees with our findings in the study. Yu et al. (2018) found that SCFA addition affected 3T3-L1 adipocyte differentiation and lipid accumulation. Correlation analysis of the results showed that acetic acid and propionic acid were negatively related to the expression of certain genes associated with adipocyte proliferation and differentiation that were detected in the study. Gabriel and Fantuzzi (2019) indicated that SCFAs were in contact with adipocytes and SCFA interventions in vivo could modulate high fat diet-induced weight gain. While another study found that folic acid influenced body weight gain under high fat feeding without changing faecal and plasma SCFAs, indicating that gut bacteria may partially share the effects of dietary folic acid on obesity, independent of SCFAs (Chen et al., 2022).

In the present study, we firstly confirmed the phenotype of abdominal fat reduction induced by dietary folic acid addition, which was supported by adipose tissue morphology and gene expression related to adipogenesis. Furthermore, consistent with the variation in bacterial genera, caecal acetic acid and propionic acid were higher in the folic acid group; and *Oscillospira* and acetic acid were identified to be negatively associated with abdominal fat percentage. Whereas folic acid itself could directly regulate the lipid metabolic pathway, it is possible that there exists another mechanism contributing to the abdominal fat lowering effect such as hepatic lipid metabolism. Undoubtedly, the findings in this study provide a new insight into the role of folic acid in regulating abdominal fat deposition mediated by gut microbiota and SCFA production. Further studies, such as *in vitro* adipocyte trials or fecal bacteria transplantation, are needed to verify this hypothesis.

## 5 Conclusion

In summary, the current research supports the view that dietary supplementation of folic acid reduces fat deposition by inhibiting abdominal adipocytes proliferation and differentiation, which might be mediated via alterations in gut microbiota and SCFAs production.

## Author contributions

Yanli Liu: experimental design, gene expression detection, Formal analysis, Writing – original draft, Funding acquisition. Jiantao Yang: animal feeding, growth performance record, Formal analysis. Yibin Wang: RNA extraction, Rui Liu: RNA extraction, Xiaoying Liu: RNA extraction, Formal analysis. Xinhuo Huang: Writing – review & editing, Yingge Li: Writing – review & editing. Ruifang Liu: manuscript revision, Writing – review & editing. Xiaojun Yang: Funding acquisition, Supervision, Writing – review & editing.

## Declaration of competing interest

We declare that we have no financial and personal relationships with other people or organizations that can inappropriately influence our work, there is no professional or other personal interest of any nature or kind in any product, service and/or company that could be construed as influencing the content of this paper.
